# Adherence to adjuvant endocrine therapy including GnRH-analogues and survival: a population-based cohort study

**DOI:** 10.1016/j.eclinm.2025.103493

**Published:** 2025-09-12

**Authors:** Louise Eriksson Bergman, Alexios Matikas, Xingrong Liu, Theodoros Foukakis

**Affiliations:** a Department of Oncology-Pathology, Karolinska Institutet, Stockholm, Sweden; b Department of Surgery and Oncology, Capio Sankt Göran Hospital, Stockholm, Sweden; c Department of Breast Cancer, Endocrine Tumors and Sarcoma, Karolinska University Hospital, Stockholm, Sweden

**Keywords:** Breast cancer, Adjuvant, Endocrine therapy, Gonadotropin-releasing hormone analogues, Adherence, Compliance, Survival, Prognosis

## Abstract

**Background:**

Adjuvant endocrine therapy (AET) combining gonadotropin-releasing hormone analogues (GnRHa) with tamoxifen (TAM) or aromatase inhibitors (AI) improves survival in premenopausal women with breast cancer compared to TAM and is increasingly prescribed. However, there are concerns regarding adherence.

**Methods:**

Through linkages to Swedish national healthcare registers, we conducted a population-based cohort study including women diagnosed with early, invasive, estrogen receptor-positive breast cancer, January 2007 to December 2020, in the Stockholm-Gotland region, Sweden, with at least one dispensation of oral AET (n = 16,468). Follow-up ended January 14, 2022.

AET was categorized based on the first dispensed oral AET (AI or TAM) and if GnRHa had been dispensed within three months of this, into the respective combination group (GnRHa + TAM or GnRHa + AI). Adherence was defined as a medication possession ratio ≥80% over five years for oral AET and two years for GnRHa, or the shortest interval from first dispensation to recurrence, contralateral breast cancer, death, emigration, or end of follow-up, if they preceded five and two years, respectively.

To study the association of type of AET and non-adherence, odds ratios (OR) were calculated using logistic regression. The association between non-adherence and invasive breast cancer-free survival (IBCFS) was studied with landmark analysis, calculating hazard ratios (HR) using the Cox proportional hazards model and flexible parametric modeling.

**Findings:**

Adherence was 86% to AI, 79% to TAM, 75% to both TAM + GnRHa, and AI + GnRHa (p < 0.001). Adjusted OR for non-adherence was 1.40 (95% confidence interval (CI) 1.27–1.55) for TAM, 2.73 (95% CI 2.19–3.40) for TAM + GnRHa, and 2.92 (95% CI 2.24–3.79) for AI + GnRHa, respectively, compared to AI. Adjusted HR of IBCFS were 1.43 (95% CI 1.26–1.64) and 1.19 (95% CI 1.04–1.35) at one- and five-year landmark analysis, respectively, comparing non-adherent to adherent groups.

**Interpretation:**

Adherence was lower to combination regimens than to single AET, and non-adherence was associated with poorer survival. Future prospective studies are warranted to validate these findings.

**Funding:**

This work was supported by grants to TF from the Swedish Cancer Society (Cancerfonden) (grant no. 21 1800Pj), Vetenskapsrådet (grant no. 2021-03061), Cancerföreningen i Stockholm (grant no. 234073), and Region Stockholm (Stockholms Läns Landsting) (grant no. FoUI-974936).

**Research in context**

**Evidence before this study**

Randomized clinical trials have found good adherence to adjuvant endocrine therapy (AET), including combinations with gonadotropin-releasing hormone analogues (GnRHa), but lack generalizability. We searched PubMed for studies from its inception to July 2025 using the search terms “adherence” or “compliance” in combination with “breast cancer” and “adjuvant” and either “endocrine therapy” or “endocrine treatment” with and without “survival” or “prognosis”. The four observational studies we found specifically investigating adherence to GnRHa were either small, lacking adherence to oral AET, or based on insurance claims and lacking information on recurrences which potentially could bias results.

**Added value of this study**

This first, truly population-based study, including 16,468 breast cancer patients and specifically investigating adherence to GnRHa in combination with oral AET, provides insights into real-world adherence patterns and its association with prognosis. The study shows a significantly lower adherence to combination regimens including GnRHa than to single treatment with either aromatase inhibitors or tamoxifen. Non-adherence was negatively associated with survival.

**Implications of all the available evidence**

An increasing number of women are being prescribed combination endocrine treatment with GnRHa in the adjuvant setting. Based on the results of this study, these combination regimens seem less well tolerated than single treatment with tamoxifen or aromatase inhibitors. The poorer survival seen in non-adherers, both in our and previous studies, highlights the need for research into factors specifically associated with adherence to combination treatment to improve support to these patients.

## Introduction

Adjuvant endocrine therapy (AET) with tamoxifen (TAM) alone is sufficient in treating premenopausal women with lowest risk of recurrence whereas combination treatments with gonadotropin-releasing hormone analogues (GnRHa) and aromatase inhibitors (AI) or GnRHa and TAM improve survival compared to TAM alone in women with higher risk.^1^ Furthermore, AET with AI is more effective than TAM in postmenopausal women.^2^^,^^3^

AETs are prescribed for 5–10 years.^4^ However, a substantial proportion of patients do not adhere to treatment^5^ and non-adherence to oral AET has been found to be associated with poorer survival.^6^^,^^7^ However, prior studies suffer from immortal time bias,^7^ possible selection bias,^5^, ^6^, ^7^ and limitations in generalizability.^5^, ^6^, ^7^

GnRHa are administered as an injection and combination treatments lead to more side-effects compared to single treatment,^8^^,^^9^ thereby possibly affecting adherence negatively. However, adherence to the treatment arms in the SOFT and TEXT trials was high in both studies: 79.8% adhered to all treatment as assigned.^10^ While randomized controlled trials exhibit high internal validity, they often lack in generalizability,^11^ which could affect treatment adherence positively in randomized clinical trials compared to the general population.

To our knowledge, no large-scale, population-based study has been performed to investigate AET adherence including combination treatments with GnRHa. Sweden has a universal health care system, and all Swedish residents have high-cost protection, severely limiting out-of-pocket costs. Furthermore, Sweden has several high quality and nearly complete national registries^12^, ^13^, ^14^, ^15^, ^16^ making it an optimal setting to study adherence. We therefore aimed to investigate adherence to AET, including GnRHa, and survival based on landmark analysis, using high-quality, real-world data in a population-based cohort.

## Methods

### Study design

This is a population- and registry-based cohort study set in the healthcare region of Stockholm–Gotland, Sweden, which accounts for 25% of the total Swedish population. Although the study was performed retrospectively, all information recorded in the registries was gathered prospectively ensuring temporality. The reporting of this study abides by the STROBE guidelines.^17^

### Study population

**Fig. 1:**
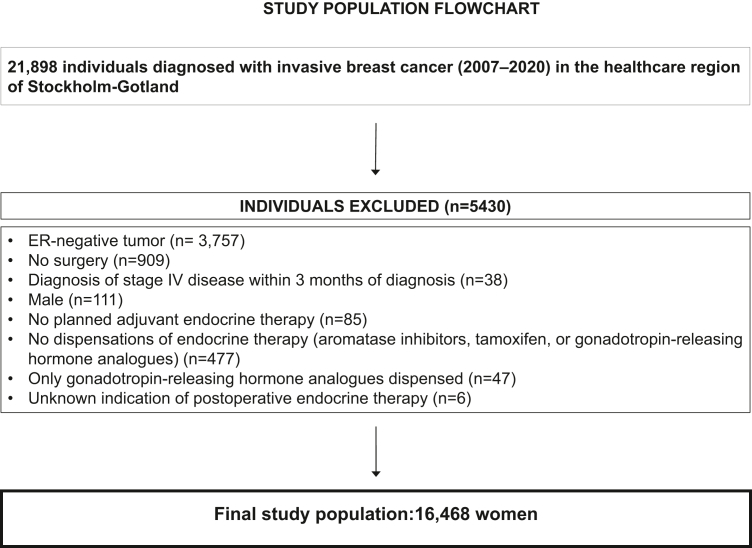
Flowchart of inclusion.

All individuals diagnosed with invasive breast cancer, 2007–2020, were identified through the National Quality Register for Breast Cancer (n = 21,898). We included all women with early, estrogen receptor-positive breast cancer who underwent surgery, were planned to receive AET, and who had at least one dispensation of oral AET (n = 16,468) (see Fig. 1).

### Ethics

Our study was approved by the Regional Ethical Review Board in Stockholm, Sweden, with reference numbers 2016/1303-31 and 2021-01147. The data analyzed were obtained from registries and informed consent of individual participants was therefore not required.

### Data sources

The National Quality Register for Breast Cancer (NKBC) includes information on diagnosis, tumor characteristics, treatment including dates of start of AET, and follow-up, and has a completeness of 98%.^13^ The Swedish Prescribed Drug Register includes nationwide information on dispensed drugs and is complete since July 1, 2005.^16^ It includes information on drug name, Anatomical Therapeutic Chemical (ATC) code, date of prescription, date of dispensation, number of packages dispensed, and number of daily doses in each package. The National Cancer Register holds information on dates of diagnosis and clinical and morphological diagnosis. Reporting to the National Cancer Register is mandatory and completeness is high.^12^ The National Patient Register attained nationwide coverage for inpatient hospitalizations in 1987. Since 2001, it is compulsory also for specialized outpatient clinics to report to the National Patient Register. Diagnoses are coded according to the International Classification of Diseases (ICD)-system. Coverage and validity of the National Patient Register is high.^14^ The Cause of Death Register covers all residents in Sweden with essentially no missing deaths^15^ and follow-up of vital status is therefore virtually complete.

Making use of the personal identity number, linkages to the Swedish Prescribed Drug Register as well as the National Cancer Register, the National Patient Register, and the Cause of Death Register were carried out, enabling us to retrieve high-quality and virtually complete information^12^^,^^14^^,^^15^ on other primary cancers, comorbidity, prescribed and dispensed drugs, vital status, and cause of death until January 14, 2022. Timeliness in reporting varies between registers. We thus had complete information on contralateral breast cancer and other primary cancers until December 31, 2020, and information on all other variables until January 14, 2022.

### Defining AET

AI, TAM, and/or GnRHa can be prescribed as fertility preserving treatment during chemotherapy. AI and TAM are then prescribed for less than one month and before the start of chemotherapy,^18^ whereas GnRHa is prescribed as fertility preservation during chemotherapy and should be initiated at least one week before start of chemotherapy.^19^ The NKBC includes start dates of adjuvant chemotherapy and AET (based on information from medical records), which enabled the extraction of fertility treatment from AET.

As principle, the start date of AET was set to the date of the first dispensation of AET according to the Prescribed Drug Register. However, in order to remove possible fertility treatment, the start date was set to the first dispensation of AET on or after the AET start date recorded in the NKBC in the following cases: Premenopausal individuals who received adjuvant chemotherapy and had a first, postoperative dispensation of AI or TAM no later than the start date of adjuvant chemotherapy (n = 52); individuals lacking a date of chemotherapy but who were ≤40 years of age, premenopausal, and had AI dispensed as first AET without GnRHa within three months (n = 9); and, lastly, individuals who had their first GnRHa dispensed either before or no later than one month after the start date of chemotherapy (to account for smaller errors in recorded chemotherapy start dates) (n = 58). Six individuals lacked information on start date of AET according to the NKBC and were thus excluded (see Fig. 1). Endocrine therapy dispensed prior to the revised AET start date was excluded.

After fertility preserving treatment had been removed, type of AET was assessed accordingly: Women were grouped into different treatment arms based on the first oral AET (AI or TAM) and if GnRHa had been dispensed within three months of this, into the respective combination group (GnRHa + TAM or GnRHa + AI).

### Calculation of adherence

Adherence was defined using the medication possession ratio (MPR) which is the total number of daily doses of medication divided by the number of days needing the medication.^20^ Oral AET was prescribed for 5–10 years whereas GnRHa was prescribed for 2–5 years during the study period. Differences in planned treatment duration are e.g. due to differences in risk of relapse, local guidelines, age, comorbidity, and changes in treatment guidelines over time. Since we did not know the planned treatment duration per individual, we calculated adherence based on the minimum duration of AET, i.e., five years for oral AET and two years for GnRHa, based on the dispensed number of daily doses of each type of AET from the first date of AET dispensation. However, if a recurrence (local/locoregional recurrence or distant metastasis), contralateral breast cancer (both in situ and invasive), death, emigration, or end of follow-up (January 14, 2022) preceded the five-year time point for oral AET or two years for GnRHa, the earliest of these dates was used as censoring date. Recurrences and contralateral breast cancers were included as censoring events since these will affect ongoing endocrine treatment. Only dispensations before the censoring date were included and in these cases the denominator used for calculating adherence was the interval from the date of first dispensation until the censoring date.

The Swedish Prescribed Drug Register includes both dispensations and returns of dispensed drugs. To correctly calculate the medication possession ratio (MPR), all returns were thus deducted from the corresponding dispensation. If an individual had preoperative TAM or AI dispensed, any surplus doses (and the corresponding number of days) were added to the postoperative intake. Since GnRHa is administered as a monthly or tri-monthly injection and can be used as fertility-preserving treatment during chemotherapy, excess GnRHa doses from preoperative treatment were not added to the postoperative intake. However, only four individuals had surplus doses from preoperative GnRHa treatment. When GnRHa is combined with TAM or AI, GnRHa can be prescribed alone during the first three months of treatment. Thus, for individuals who started with GnRHa as first AET, the daily doses of GnRHa until the first dispensation of either TAM or AI were included in the calculation of the MPR for oral AET, up to a maximum of three months.

In line with previous studies, adherence was defined as an MPR of ≥80%.^5^ For individuals on combination treatment, patients had to have an MPR ≥ 80% to both oral AET and GnRHa to be adherent.

### Recurrences

Information on local, locoregional, and distant recurrences is recorded in the NKBC and routine follow-up of recurrence variables is performed at 5 and 10 years after diagnosis, respectively.^21^ Local and locoregional recurrences are discussed at multidisciplinary conferences regarding operability, after which the recurrences are reported to the NKBC. However, these routines do not usually apply for distant recurrences since most cases will not be eligible for surgery. Thus, to improve completeness regarding distant recurrences, we made use of the National Patient Register, National Cancer Register, and Cause of Death Register and added diagnoses of distant metastasis if any of the following criteria were met: The patient had a diagnosis of distant metastasis recorded on at least two occasions and had no other cancer other than breast cancer prior to this; the patient had one recorded diagnosis of distant metastasis but died within one year of this date and had no other cancer other than breast cancer recorded at any time; or the patient had one recorded diagnosis of distant metastasis, had no other cancer than breast cancer, and died from breast cancer. The last category was included to not miss individuals who for example sought alternative treatment.

### Covariates

Information on age, menopausal status, tumor stage, treating hospital, and treatment was extracted from the NKBC. Missingness was low: Age (0%); year of diagnosis (0%); TNM stage (3.3%); chemotherapy (<0.1%, n = 41); and adjuvant radiotherapy (<0.1%, n = 46). In total, 192 individuals (1%) had no information on menopausal status recorded and another 359 individuals (2%) had unknown menopausal status recorded, e.g. due to previous hysterectomy. Furthermore, there may be erroneous recordings and menopausal status may change from pre-/perimenopausal to postmenopausal after chemotherapy due to chemotherapy-induced ovarian toxicity.^22^ Thus, menopausal status was recoded based on AET dispensation (after fertility treatment had been removed from AET) as follows: Individuals >60 years of age and who were not postmenopausal according to the NKBC were reclassified as postmenopausal (n = 155); individuals who had GnRHa dispensed within three months of start of AET, and who were not classified as premenopausal according to the NKBC were reclassified as premenopausal (n = 72); lastly, individuals who had AI dispensed as first treatment without a GnRHa dispensation within three months and who were not postmenopausal according to NKBC were reclassified as postmenopausal (n = 519).

During the study period, the region of Stockholm–Gotland had a maximum of five hospitals with oncological clinics. Three were active during the whole study period whereas Danderyd's hospital was active in the early part and Capio S:t Görans Hospital in the latter. Patients are distributed to the different hospitals based on catchment area. All of the hospitals use the same regional therapy guidelines for adjuvant therapy.

The Charlson Comorbidity Index (CCI) was calculated in accordance with the study by Ludvigsson et al.^23^ We further extracted information on the following disorders prior to start of AET that have similar symptoms to or are known side-effects of AET and which could confound the relationship between different AET regimens and adherence: Depression (F32, F33), anxiety (F40, F41), osteoporosis (M80, M81, M82), rheumatic disease (M05, M06, M070–M073, M08, M123, M13, M30, M313–M316, M32–M34, M350–M353, M45, and M46), urinary tract disorders (N30, N34, N39), and gynecological disorders (N70–N90, N94). Prior deep vein thrombosis was not included since TAM is contraindicated for these individuals.

In analyses, covariates were treated as they are presented in Table 1 except for CCI which was recategorized as a binary variable (CCI = 0 or CCI ≥ 1).

**Table 1:** Baseline characteristics based on first dispensed adjuvant endocrine therapy; aromatase inhibitors (AI), tamoxifen (TAM), TAM + gonadotropin-releasing hormone analogues (GnRHa), or AI + GnRHa.

	First dispensed adjuvant endocrine therapy				
	AI (n = 8336)	TAM (n = 7121)	TAM + GnRHa (n = 632)	AI + GnRHa (n = 379)	Total (n = 16,468)
**Age at diagnosis**	67 (30–97)	58 (21–94)	41 (21–54)	45 (25–57)	62 (21–97)
**Year of diagnosis**					
2007–2014	3983 (48%)	4860 (68%)	226 (36%)	62 (16%)	9131 (55%)
2015–2017	2087 (25%)	1442 (20%)	150 (24%)	73 (19%)	3752 (23%)
2018–2020	2266 (27%)	819 (12%)	256 (41%)	244 (64%)	3585 (22%)
**Menopausal status**					
Premenopausal	0 (0%)	2692 (38%)	632 (100%)	379 (100%)	3703 (22%)
Postmenopausal	8336 (100%)	4203 (59%)	0 (0%)	0 (0%)	12,539 (76%)
Unknown	0 (0%)	226 (3%)	0 (0%)	0 (0%)	226 (1%)
**Charlson comorbidity index**					
0	5056 (61%)	5401 (76%)	561 (89%)	313 (83%)	11,331 (69%)
1	2270 (27%)	1334 (19%)	62 (10%)	57 (15%)	3723 (23%)
≥2	1010 (12%)	386 (5%)	9 (1%)	9 (2%)	1414 (9%)
**Rheumatic disease**^a^					
Yes	444 (5%)	282 (4%)	10 (2%)	9 (2%)	745 (5%)
No	7892 (95%)	6839 (96%)	622 (98%)	370 (98%)	15,723 (95%)
**History of depression/anxiety**^b^					
Yes	758 (9%)	592 (8%)	83 (13%)	56 (15%)	1489 (9%)
No	7578 (91%)	6529 (92%)	549 (87%)	323 (85%)	14,979 (91%)
**History of urinary tract infections/urethritis**^c^					
Yes	1518 (18%)	974 (14%)	93 (15%)	67 (18%)	2652 (16%)
No	6818 (82%)	6147 (86%)	539 (85%)	312 (82%)	13,816 (84%)
**History of gynecological disorders**^d^					
Yes	2049 (25%)	1761 (25%)	262 (41%)	170 (45%)	4242 (26%)
No	6287 (75%)	5360 (75%)	370 (59%)	209 (55%)	12,226 (74%)
**Osteoporosis**^e^					
Yes	284 (3%)	191 (3%)	0 (0%)	0 (0%)	475 (3%)
No	8052 (97%)	6930 (97%)	632 (100%)	379 (100%)	15,993 (97%)
**TNM stage**^f^					
1	3188 (38%)	4795 (67%)	137 (22%)	73 (19%)	8193 (50%)
2	3991 (48%)	1849 (26%)	387 (61%)	231 (61%)	6458 (39%)
3	871 (10%)	247 (3%)	96 (15%)	66 (17%)	1280 (8%)
Unknown	286 (3%)	230 (3%)	12 (2%)	9 (2%)	537 (3%)
**HER2-status**					
Positive	1049 (13%)	456 (6%)	174 (28%)	69 (18%)	1748 (11%)
Negative	7043 (85%)	6386 (90%)	454 (72%)	305 (80%)	14,188 (86%)
Unknown	244 (3%)	279 (4%)	4 (1%)	5 (1%)	532 (3%)
**Treating hospital**					
Capio S:t Görans Hospital	1044 (13%)	351 (5%)	120 (19%)	94 (25%)	1609 (10%)
Södersjukhuset	3243 (39%)	3301 (46%)	429 (68%)	131 (35%)	7104 (43%)
Karolinska University Hospital	3196 (38%)	2525 (35%)	71 (11%)	145 (38%)	5937 (36%)
Danderyd's Hospital	643 (8%)	807 (35%)	5 (1%)	6 (2%)	1461 (9%)
Visby Lasarett/other^g^	210 (3%)	137 (2%)	7 (1%)	3 (1%)	357 (2%)
**Neoadjuvant treatment**					
Yes	934 (11%)	407 (6%)	219 (35%)	189 (50%)	1749 (11%)
No	7391 (89%)	6709 (94%)	413 (65%)	190 (50%)	14,703 (89%)
Unknown	11 (0%)	5 (0%)	0 (0%)	0 (%)	16 (0%)
**Type of breast surgery**					
No breast surgery	12 (0%)	2 (0%)	0 (0%)	1 (0%)	15 (0%)
Partial mastectomy	5303 (64%)	5253 (74%)	284 (45%)	184 (49%)	11,024 (67%)
Mastectomy	3021 (36%)	1865 (26%)	348 (55%)	194 (51%)	5428 (33%)
Unknown	0 (0%)	1 (0%)	0 (0%)	0 (0%)	1 (0%)
**Type of axillary surgery**					
No axillary surgery	114 (1%)	97 (1%)	2 (0%)	2 (1%)	215 (1%)
Sentinel node biopsy and/or sampling	5200 (62%)	5785 (81%)	304 (48%)	171 (45%)	11,460 (70%)
Axillary lymph node dissection	3015 (36%)	1238 (17%)	326 (52%)	206 (54%)	4785 (29%)
Unknown	7 (0%)	1 (0%)	0 (0%)	0 (0%)	8 (0%)
**Chemotherapy** (neoadjuvant and/or adjuvant)					
Yes	3757 (45%)	1778 (25%)	573 (91%)	333 (88%)	6441 (39%)
No	4557 (55%)	5325 (75%)	58 (9%)	46 (12%)	9986 (61%)
Unknown	22 (0%)	18 (0%)	1 (0%)	0 (0%)	41 (0%)
**Radiotherapy**					
Yes	6580 (79%)	5806 (82%)	532 (84%)	350 (92%)	13,268 (81%)
No	1734 (21%)	1301 (18%)	91 (14%)	28 (7%)	3154 (19%)
Unknown	22 (0%)	14 (0%)	9 (1%)	1 (0%)	46 (0%)

a ICD codes M05, M06, M070–M073, M08, M123, M13, M30, M313–M316, M32–M34, M350–M353, M45, and M46.

b ICD codes F32, F33, F40, and F41.

c ICD codes N30, N34, and N39.

d ICD codes N70–N90 and N94.

e ICD codes M80, M81, and M82.

f TNM stage is based on clinical stage for individuals treated with neoadjuvant therapy and pathological stage for individuals who had primary surgery.

g 223 patients were treated at Visby Lasarett, Gotland. The remaining 134 individuals were treated in other parts of Sweden.

### Statistics

Delays in treatment initiation were calculated using the date of prescription and date of dispensation recorded in the Prescribed Drug Register. Univariate analysis of adherence, delays, and switches based on type of AET was performed using the Chi^2^ test.

Logistic regression was used to study the association between AET and non-adherence adjusting for age, menopausal status (except in stratified analysis on menopausal status), CCI, history of depression/anxiety, history of urinary tract disorders, history of gynecological disorders, history of rheumatic disorders, TNM stage, neoadjuvant/adjuvant chemotherapy, adjuvant radiotherapy, treating hospital, and year of diagnosis, and stratifying on menopausal status. In this analysis, we were interested in the overall effect of initial AET type on risk of non-adherence since the first dispensed type of adjuvant endocrine therapy will be the most optimal treatment for the patient based on risk of recurrence and menopausal status at that time point. Although switching oral treatments may be associated with non-adherence, switches occur after initial AET assignment and are thus post-exposure events. Switches may therefore be mediators in the causal pathway between type of AET and non-adherence but cannot be confounders and were consequently not adjusted for.

In survival analysis, we aimed to investigate non-adherence and its association with invasive breast cancer-free survival (IBCFS).^24^ To mitigate immortal time bias, landmark analysis at one, two, three, four and five years after first dispensation of AET was conducted. For the one-year landmark, only individuals who had at least one year of follow-up from date of first dispensation of AET and thus no recurrence (local, regional, or distant), contralateral breast cancer, death, or emigration during this time were included. For landmarks two to five years, the same applied as above but with changing landmark time accordingly. Adherence to AET was determined from start of AET to each respective landmark. Start of follow-up of survival was from each landmark, respectively; for example, at landmark one year, follow-up started at one year after dispensation. IBCFS was defined as time from each specific yearly landmark after the first AET dispensation to occurrence of one of the following events: local, locoregional, or distant recurrence, contralateral invasive breast cancer, or death from any cause, whichever occurred first.^24^ Women were censored if they did not experience any of the above events at the end of follow-up or were lost to follow-up because of emigration.

**Fig. 2:**
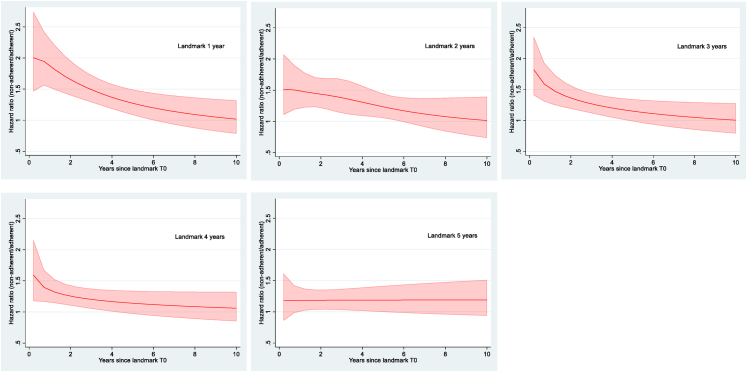
Association between adherence and invasive breast cancer-free survival (IBCFS) using flexible parametric survival modeling for landmark time of 1 year, 2 years, 3 years, 4 years, and 5 years, respectively, after first dispensation of adjuvant endocrine therapy. All models are adjusted for age, menopausal status, CCI, diagnosis of depression/anxiety before start of adjuvant endocrine therapy, TNM stage, neoadjuvant/adjuvant chemotherapy, adjuvant radiotherapy, and year of diagnosis.

Survival analysis was performed using the Cox proportional hazards model to calculate hazard ratios (HR). However, the assumption of proportionality over time was not met and we therefore also conducted flexible parametric modeling.^25^ For time-dependent effects we assessed goodness of fit of models with different degrees of freedom using the Akaike Information Criterion^26^ and chose the model with best fit for each landmark (see Fig. 2). The adjusted model included age, menopausal status (except in stratified analysis), CCI, diagnosis of depression/anxiety before start of AET, TNM stage, neoadjuvant/adjuvant chemotherapy, adjuvant radiotherapy, treating hospital, and year of diagnosis and stratifying on menopausal status. We did not include type of AET due to the potential issue of collinearity between type of AET and menopausal status. A possible interaction between adherence and menopausal status was studied by comparing the fits of models with and without the interaction term using the likelihood ratio test.

All analyses were performed using the statistical software, STATA 16.1 (StataCorp LP, College Station, TX, USA).

### Role of the funding source

The study did not receive any specific funding. Funding sources that support the authors stated in the acknowledgement section did not have any role in study inception, data collection, analysis, interpretation, writing of the manuscript or the decision to submit. The corresponding author had full access to all the data in the study and had final responsibility for the decision to submit for publication.

## Results

### Patient characteristics

Patients' clinical and demographic characteristics are presented in Table 1. Type of first treatment with AET was distributed as follows: 8336 women (50.6%) had AI only dispensed; 7121 women (43.2%) had TAM only, of whom 2692 were premenopausal; 632 women (3.8%) had TAM and GnRHa dispensed; and 379 (2.3%) had AI and GnRHa dispensed. 12,539 individuals were postmenopausal, 3703 premenopausal, and 226 had unknown menopausal status. As expected, individuals in the combination groups were younger, healthier (except for a higher prevalence of depression/anxiety), had more advanced tumor stage, more often HER2-positive disease, and received more extensive treatment than individuals in the single treatment groups.

### Adherence to treatment

Delay in treatment initiation was uncommon in all groups but more frequently occurred in the AI-only and AI + GnRHa groups (3% in both) compared to TAM + GnRHa and TAM-only (2% in both) (p < 0.001) (Table 2). Switches between oral treatments was least common in the TAM + GnRHa group with 13% compared to 18% for TAM-only, 19% for AI + GnRHa, and 20% for AI-only (p < 0.001) (Table 2).

**Table 2:** Delays, switches and adherence based on type of first dispensed adjuvant endocrine therapy (aromatase inhibitors (AI), tamoxifen (TAM), TAM + gonadotropin-releasing hormone analogues (GnRHa), and AI + GnRHa).

	AI (n = 8336)	TAM (n = 7121)	TAM + GnRHa (n = 632)	AI + GnRHa (n = 379)	Total (n = 16,468)	p-value
**Initiation delay >3 months**						<0.001
Yes	220 (3%)	108 (2%)	11 (2%)	10 (3%)	349 (2%)	
No	8116 (97%)	7013 (98%)	621 (98%)	369 (97%)	16,119 (98%)	
**Switch between oral treatment (within 5 years)**^a^						<0.001
Yes	1627 (20%)	1252 (18%)	82 (13%)	72 (19%)	3033 (18%)	
No	6709 (80%)	5869 (82%)	550 (87%)	307 (81%)	13,435 (82%)	
**Switch between oral treatment (within 5 years)**^a^**only including premenopausal women**						0.036
Yes	NA	418 (16%)	82 (13%)	72 (19%)	572 (15%)	
No	NA	2274 (84%)	550 (87%)	307 (81%)	3131 (85%)	
**MPR ≥ 0.8 for TAM/AI**^b^						<0.001
Adherent	7146 (86%)	5657 (79%)	527 (83%)	336 (89%)	13,666 (83%)	
Non-adherent	1190 (14%)	1464 (21%)	105 (17%)	43 (11%)	2802 (17%)	
**MPR ≥ 0.8 for TAM/AI**^b^**only including premenopausal women**						<0.001
Adherent	NA	2158 (80%)	527 (83%)	336 (89%)	3021 (82%)	
Non-adherent	NA	534 (20%)	105 (17%)	43 (11%)	682 (18%)	
**MPR ≥ 0.8 for GnRHa**^c^						0.117
Adherent	NA	NA	545 (86%)	313 (83%)	858 (85%)	
Non-adherent	NA	NA	87 (14%)	66 (17%)	153 (15%)	
**MPR ≥ 0.8 for all treatment**^d^						<0.001
Adherent	7146 (86%)	5657 (79%)	472 (75%)	283 (75%)	13,558 (82%)	
Non-adherent	1190 (14%)	1464 (21%)	160 (25%)	96 (25%)	2910 (18%)	
**MPR ≥ 0.8 for all treatment**^d^**only including premenopausal women**						0.001
Adherent	NA	2158 (80%)	472 (75%)	283 (75%)	2913 (79%)	
Non-adherent	NA	534 (20%)	160 (25%)	96 (25%)	790 (21%)	

a Includes switches from TAM to AI and vice versa. Switches between different AI:s are not included.

b Medication possession ratio (MPR) ≥0.8 based on total pill dispensation of TAM/AI including dispensations of GnRHa up to 3 months if GnRHa dispensation preceded dispensation of TAM or AI.

c MPR ≥ 0.8 based on dispensation of GnRHa.

d MPR ≥ 0.8 based on both pill and GnRHa dispensation such that adherence requires both adherence to AI/TAM and adherence to GnRHa if the individual has combination treatment, but only adherence to AI/TAM if the individual only has AI/TAM.

Adherence to oral AET ranged from 79% in the TAM-only group to 89% in the AI + GnRHa group (<0.001) (Table 2). Restricting analysis to premenopausal women, adherence patterns were similar to oral AET. In total, 153 individuals were non-adherent to GnRHa (Table 2). Of these individuals, 29% (45 individuals) were also non-adherent to oral AET. Basing adherence on both oral AET and GnRHa decreased adherence to 75% in both combination groups (p < 0.001) (Table 2).

Logistic regression yielded an adjusted OR of non-adherence of 1.40 (95% CI 1.27–1.55) for TAM, 2.73 (95% CI 2.19–3.40) for TAM + GnRHa, and 2.92 (95% CI 2.24–3.79) for AI + GnRHa, respectively, compared to AI only (Table 3). Restricting analysis to premenopausal women yielded an adjusted OR of 1.60 (95% CI 1.25–2.04) for TAM + GnRHa and 1.80 (95% CI 1.35–2.40) for AI + GnRHa, respectively, compared to TAM only (Table 3).

**Table 3:** Association between types of adjuvant endocrine therapy (aromatase inhibitors (AI), tamoxifen (TAM), TAM + gonadotropin-releasing hormone analogues (GnRHa), and AI + GnRHa) and non-adherence in all women and stratified on menopausal status.

Type of endocrine therapy	All			Postmenopausal			Premenopausal		
	Odds ratio (OR) of non-adherence	95% CI	p-value	OR of non-adherence	95% CI	p-value	OR of non-adherence	95% CI	p-value
**Crude model**	**n = 16,468**			**n = 12,539**			**n = 3703**		
AI	1.00 (Ref.)			1.00 (Ref.)			NA		
TAM	1.55	1.43–1.69	<0.001	1.62	1.47–1.78	<0.001	1.00 (Ref.)		
TAM + GnRHa	2.04	1.68–2.46	<0.001	NA			1.37	1.12–1.68	0.002
AI + GnRHa	2.04	1.60–2.59	<0.001	NA			1.37	1.07–1.76	0.013
**Full model**^a^	**n = 16,391**			**n = 12,482**			**n = 3683**		
AI	1.00 (Ref.)			1.00 (Ref.)			NA		
TAM	1.40	1.27–1.55	<0.001	1.31	1.17–1.47	<0.001	1.00 (Ref.)		
TAM + GnRHa	2.73	2.19–3.40	<0.001	NA			1.60	1.25–2.04	<0.001
AI + GnRHa	2.92	2.24–3.79	<0.001	NA			1.80	1.35–2.40	<0.001

a Adjusted for age, menopausal status, Charlson Comorbidity Index, history of urinary tract disorder, history of depression/anxiety, history of gynecological disorder, history of rheumatic disease, TNM stage, treating hospital, neoadjuvant/adjuvant chemotherapy, adjuvant radiotherapy, and year of diagnosis. When limiting analysis to premenopausal women, the same model was used but removing menopausal status.

The poorer adherence to combination treatments could be due to ongoing toxicity of previously received chemotherapy. Analysis of AET and risk of non-adherence was adjusted for chemotherapy, and the associations seen should thus be independent of this. However, to specifically explore this hypothesis, we conducted a post-hoc analysis restricted to premenopausal women and stratifying on chemotherapy (adjuvant/neoadjuvant chemotherapy versus no chemotherapy). We found that the most pronounced association between combination treatments and non-adherence was actually seen in the group that did not receive chemotherapy although confidence intervals overlapped (Supplementary Table S1). Thus, the poorer adherence to combination treatments cannot be explained by previously received chemotherapy.

### Survival analysis

Adherence in multiple landmark analyses, i.e., separately only assessing adherence in individuals with no events and with full follow-up at each respective timepoint, showed that adherence to oral AET dropped from 94% to 81% at landmark time of one year and five years, respectively (Supplementary Figure S1). Comparing the same landmark time points, adherence dropped from 83% to 70% in women with combination treatments (Supplementary Figure S1).

Although the association between non-adherence and IBCFS was non-proportional, i.e., varied over follow-up time, the HRs obtained from the Cox model were statistically significant and can thus be interpreted as the weighted average of time-varying HRs.^27^ In the one-year landmark analysis, the adjusted HR was 1.43 (95% CI 1.26–1.63) and decreased in the consequential analyses of landmark times but was still elevated at landmark five years with an adjusted HR of 1.19 (95% CI 1.04–1.36) (Table 4). Analyses stratified on menopausal status showed similar results for both groups. Furthermore, there was no evidence of an interaction between adherence and menopausal status (p = 0.7906, p = 0.7047, p = 0.5555, p = 0.4061, and p = 0.5876 for landmark one year, two years, three years, four years, and five years, respectively). Since we lacked information on contralateral breast cancer after December 31, 2020, sensitivity analysis was performed to ensure robustness of results from the main analyses based on patients who had first date of dispensation more than five years prior to this date and setting December 31, 2020, as end of follow-up. Results and conclusions remained unchanged.

**Table 4:** Adherence to adjuvant endocrine therapy and invasive breast cancer-free survival (IBCFS)^a^ using landmark analyses at 1 year, 2 years, 3 years, 4 years, and 5 years, respectively.

	All			Postmenopausal			Premenopausal		
**1 year landmark**									
Descriptive statistics	**N**	**Follow-up (fu) person years**	**Events (n)**	**N**	**Fu person years**	**Events (n)**	**N**	**Fu person years**	**Events (n)**
Adherent	14,643	84,876	2464	11,231	64,629	2136	3204	18,906	310
Non-adherent	1063	5470	254	733	3812	212	318	1597	39
Crude	**HR**	**95% CI**	**p-value**	**HR**	**95% CI**	**p-value**	**HR**	**95% CI**	**p-value**
Adherent	1.00 (Ref.)			1.00 (Ref.)			1.00 (Ref.)		
Non-adherent	1.61	1.42–1.83	<0.001	1.70	1.48–1.96	<0.001	1.48	1.06–2.06	0.021
Adjusted^b^									
Adherent	1.00 (Ref.)			1.00 (Ref.)			1.00 (Ref.)		
Non-adherent	1.43	1.26–1.63	<0.001	1.39	1.20–1.61	<0.001	1.43	1.02–2.00	0.039
**2 year landmark**									
Descriptive statistics	**N**	**Fu person years**	**Events (n)**	**N**	**Fu person years**	**Events (n)**	**N**	**Fu person years**	**Events (n)**
Adherent	12,673	67,642	2020	9732	51,421	1757	2762	15,139	251
Non-adherent	1573	7649	331	1166	5544	285	380	1997	41
Crude	**HR**	**95% CI**	**p-value**	**HR**	**95% CI**	**p-value**	**HR**	**95% CI**	**p-value**
Adherent	1.00 (Ref.)			1.00 (Ref.)			1.00 (Ref.)		
Non-adherent	1.46	1.30–1.64	<0.001	1.52	1.34–1.72	<0.001	1.23	0.88–1.71	0.225
Adjusted^b^									
Adherent	1.00 (Ref.)			1.00 (Ref.)			1.00 (Ref.)		
Non-adherent	1.31	1.17–1.48	<0.001	1.30	1.14–1.47	<0.001	1.21	0.86–1.69	0.274
**3 year landmark**									
Descriptive statistics	**N**	**Fu person years**	**Events (n)**	**N**	**Fu person years**	**Events (n)**	**N**	**Fu person years**	**Events (n)**
Adherent	10,834	53,976	1639	8323	41,051	1444	2345	12,041	183
Non-adherent	1723	7903	339	1257	5667	291	438	2130	44
Crude	**HR**	**95% CI**	**p-value**	**HR**	**95% CI**	**p-value**	**HR**	**95% CI**	**p-value**
Adherent	1.00 (Ref.)			1.00 (Ref.)			1.00 (Ref.)		
Non-adherent	1.42	1.26–1.60	<0.001	1.47	1.30–1.67	<0.001	1.34	0.97–1.87	0.078
Adjusted^b^									
Adherent	1.00 (Ref.)			1.00 (Ref.)			1.00 (Ref.)		
Non-adherent all	1.31	1.16–1.47	<0.001	1.27	1.12–1.45	<0.001	1.29	0.92–1.81	0.135
**4 year landmark**									
Descriptive statistics	**N**	**Fu person years**	**Events (n)**	**N**	**Fu person years**	**Events (n)**	**N**	**Fu person years**	**Events (n)**
Adherent	9393	42,489	1324	7267	32,367	1175	1979	9416	138
Non-adherent	1757	7523	309	1262	5285	262	466	2140	42
Crude	**HR**	**95% CI**	**p-value**	**HR**	**95% CI**	**p-value**	**HR**	**95% CI**	**p-value**
Adherent	1.00 (Ref.)			1.00 (Ref.)			1.00 (Ref.)		
Non-adherent	1.32	1.17–1.50	<0.001	1.37	1.20–1.57	<0.001	1.33	0.94–1.87	0.108
Adjusted^b^									
Adherent	1.00 (Ref.)			1.00 (Ref.)			1.00 (Ref.)		
Non-adherent all	1.24	1.10–1.41	0.001	1.20	1.05–1.38	0.009	1.27	0.89–1.81	0.180
**5 year landmark**									
Descriptive statistics	**N**	**Fu person years**	**Events (n)**	**N**	**Fu person years**	**Events (n)**	**N**	**Fu person years**	**Events (n)**
Adherent	7808	32,441	1051	6026	24,647	938	1661	7254	103
Non-adherent	1819	7210	294	1311	5105	253	480	2001	35
Crude	**HR**	**95% CI**	**p-value**	**HR**	**95% CI**	**p-value**	**HR**	**95% CI**	**p-value**
Adherent	1.00 (Ref.)			1.00 (Ref.)			1.00 (Ref.)		
Non-adherent	1.26	1.11–1.43	<0.001	1.30	1.14–1.50	<0.001	1.22	0.83–1.80	0.303
Adjusted^b^									
Adherent	1.00 (Ref.)			1.00 (Ref.)			1.00 (Ref.)		
Non-adherent all	1.19	1.04–1.36	0.009	1.16	1.00–1.33	0.044	1.20	0.81–1.78	0.364

a IBCFS includes local recurrence, regional recurrence, contralateral invasive breast cancer, distant metastasis, and death due to any cause.

b Adjusted for age, the Charlson Comorbidity Index, diagnosis of depression/anxiety before start of AET, year of diagnosis, TNM stage, treating hospital, neoadjuvant/adjuvant chemotherapy (yes/no), and adjuvant radiotherapy (yes/no). In the analysis of the full cohort, menopausal status was also adjusted for.

Fig. 2 depicts time-varying HRs obtained from flexible parametric modeling for each landmark timepoint based on the fully adjusted model. For landmark timepoints of one to four years after first AET dispensation, the highest HRs were seen immediately after the landmark time and then decreased with time. For landmark timepoint five years, the shape of HRs differed, showing an approximate constant HR of 1.2.

## Discussion

In this population-based study, we found that adherence to single treatment with AI and TAM was high, and, when considered separately, adherence to oral treatment and GnRHa was also high in women prescribed combination treatment. However, adherence decreased when adherence to both oral AET and GnRHa was required. This distinction is clinically important since single treatment with oral AET or GnRHa is insufficient in reducing recurrence risk in all but the lowest risk group. After adjusting for known confounders, risk of non-adherence was almost three times as high for combination treatment compared to AI only. When restricting analysis to premenopausal women, risk of non-adherence was almost doubled when comparing combination treatments to TAM only. The lower adherence to combination treatments is especially concerning since individuals prescribed combination regimens also have high risk disease, and we found that non-adherence was associated with poorer prognosis.

Benefits of TAM and AI:s persist after cessation; for example, five years treatment with TAM will reduce risk of recurrence up to five years after cessation.^28^ Since combination therapy became more common during the latter part of the study period, and thus had the shortest follow-up, we may not be able to show the full impact of non-adherence on survival.

Adherence rates to oral AET have varied between previous studies from 41 to 88% for TAM and 50 to 91% for AI.^5^ Decreased costs for AET have been found to be effective in promoting adherence.^29^ Sweden's universal health care system, high-cost protection, and the consistent use of generic formulations to decrease costs may explain the relatively high adherence rates we found.

In non-combination treatment groups, we found that adherence was higher to AI than TAM which is in line with most previous studies.^30^ Since combination treatments are associated with higher toxicity than TAM alone and the injection of GnRHa is also associated with some discomfort, we believe that our finding of a lower adherence to combination treatment is probable. Few previous studies have assessed adherence to combination treatments^31^, ^32^, ^33^ and, to our knowledge, our study is the first population-based study to investigate adherence to combination treatments. In the largest study to date by Reeder-Hayes et al., approximately half of patients discontinued AET before five years, but the proportions were not affected by the addition of GnRHa.^31^ However, treatment information was based on insurance claims, mean follow-up time was only 1.55 years, and the study further lacked information on recurrences which could bias findings. As in the study by Sukumar et al.,^32^ non-adherence to GnRHa may partially be explained by women undergoing bilateral oophorectomy instead. Since adherence to GnRHa in our study was based on two years, the surgical procedure must have been performed within two years of first AET dispensation to affect adherence. Furthermore, the surgical procedure is relatively rare in Sweden; according to Sundell et al.,^34^ only 543 oophorectomies were performed in individuals 15–44 years of age with any prior malignant diagnosis between 2005 and 2020 in all of Sweden. Hence, we do not believe that oophorectomies can explain the lower adherence to combination treatments in our study.

In line with the previous studies by Hershman et al.^7^ and Dumas et al.^6^ on adherence to oral AET and prognosis, we also found that non-adherence was associated with decreased survival. We found that the highest increase in recurrence/death was at landmark time one year and decreased with each subsequent year. This pattern is biologically plausible—i.e., that a poor adherence close to diagnosis is more detrimental than poor adherence later.

This study has several strengths including study size, the population-based design, and almost complete coverage of the registries used including the Prescribed Drug Register to ascertain adherence. Registry-based prescription data has been found to be the most objective measure of adherence when studying large populations over longer periods of time.^35^ The findings of this study are thus generalizable to countries with similar healthcare systems, whereas adherence may be even lower in countries with non-universal healthcare systems and/or where AET costs are higher.

A limitation of this study was that we lacked information on the intended treatment duration of AET. We thus assessed adherence based on the shortest possible treatment duration of adjuvant AI/TAM and GnRHa - five and two years, respectively. Since adherence declined during follow-up, the proportion of adherent individuals may be even lower for longer intended treatment. This is clinically highly relevant, since many guidelines now recommend five years of GnRHa based on the results of the TEXT and SOFT trials.^36^ Furthermore, since adherence was based on dispensations, there may have been individuals prescribed combination therapy with TAM who never started GnRHa. We also excluded 47 individuals who only had GnRHa dispensed since we could not assign them to a particular treatment group. As in the example of treatment duration, both these two cases could only lead to an overestimation of adherence, specifically in the combination groups. Our finding of a low adherence in individuals with combination therapy will therefore, if anything, be an underestimation.

Pistilli et al. compared self-reported adherence to serum levels of TAM and found that self-reported adherence was higher.^37^ Similarly, adherence based on dispensation data may overestimate adherence since dispensations do not perfectly reflect medication intake. However, we believe that it is the best available proxy in order to study adherence in large, population-based studies, and could only overestimate adherence.

We further lacked information on BMI and socioeconomic status (SES). Since neither SES nor BMI affects type of AET prescribed, confounding could only be present in the analysis of adherence and survival. However, BMI has been shown to be positively associated with compliance^38^^,^^39^ and negatively associated with prognosis.^40^ Thus, if there is residual confounding by BMI, this would only have weakened the association seen between adherence and survival. In a systematic review of determinants of adherence to AET, SES was only to a lesser extent found to be associated with adherence.^41^ Furthermore, a Danish, population-based study investigating TAM discontinuation within five years and breast cancer recurrence found that there was little confounding by household income, marital status and education.^42^ Given the similarities between the healthcare systems in Denmark and Sweden, we believe that any confounding by SES should be small.

Finally, the region of Stockholm–Gotland is diverse pertaining to foreign birthplace, making our results generalizable to other heterogeneous settings. However, research is needed to investigate whether the results from this study are affected by ethnicity.

The register-based design of this study allows for analysis of adherence in a large population but cannot answer the question why individuals stop adhering to treatment. Decreased costs for AET, increased support in managing side-effects, reminder interventions, exercise, and reducing negative attitudes towards AET may all improve adherence to AET.^29^ However, most previous studies investigating factors associated with adherence were based on adherence to either TAM or AI. Since combination treatments have been found to be more efficacious than TAM alone in improving survival in premenopausal women, they are more commonly being prescribed. In light of the lower adherence to combination regimens found in our study and especially considering that non-adherence was associated with poorer survival, future studies are urgently needed to examine factors specifically associated with adherence to combination treatments to improve adherence and thus survival. Lastly, with the introduction of adjuvant treatment with CDK4/6-inhibitors in combination with AET in both pre- and postmenopausal women, future studies are warranted to assess the impact of CDK4/6-inhibitors on adherence.

## Contributors

L.E.B. and T.F. conceived the study. All authors designed the study and provided critical input. L.E.B. managed data collection, extracted and managed the data. L.E.B. and T.F. had direct access to and verified the underlying data. L.E.B. carried out the data analysis with help from X.L. All authors helped in the interpretation of data. L.E. wrote the first draft of the article which all authors critically reviewed. All authors approved the final draft.

## Data sharing statement

The dataset supporting the findings of this study is available from the National Quality Register for Breast Cancer (NKBC), the Swedish Prescribed Drug Register, the National Cancer Register, the National Patient Register, and the Cause of Death Register upon request.

## Declaration of interests

Alexios Matikas: Consultancy/speaker (no personal fees): Veracyte, Seagen, Roche, AstraZeneca. Research funding paid to institution: MSD, AstraZeneca, Novartis, Veracyte. Advisory board (no compensation): Nordic Pharma.

Theodoros Foukakis: Institutional fees for consultancy to Astra Zeneca, Daiichi Sankyo, Novartis, and Roche; honoraria from UpToDate; research funding to institution from AstraZeneca, Novartis, and Veracyte.

Louise Eriksson Bergman and Xingrong Liu report no conflicts of interest.

## References

[1] Francis P.A., Fleming G.F., Lang I. (2023). Adjuvant endocrine therapy in premenopausal breast cancer: 12-year results from SOFT. J Clin Oncol.

[2] Early Breast Cancer Trialists' Collaborative Group (EBCTCG) (2015). Aromatase inhibitors versus tamoxifen in early breast cancer: patient-level meta-analysis of the randomised trials. Lancet.

[3] Papakonstantinou A., Villacampa G., Navarro V. (2025). Adjuvant endocrine treatment strategies for non-metastatic breast cancer: a network meta-analysis. EClinicalMedicine.

[4] Burstein H.J., Lacchetti C., Anderson H. (2019). Adjuvant endocrine therapy for women with hormone receptor-positive breast cancer: ASCO clinical practice guideline focused update. J Clin Oncol.

[5] Murphy C.C., Bartholomew L.K., Carpentier M.Y., Bluethmann S.M., Vernon S.W. (2012). Adherence to adjuvant hormonal therapy among breast cancer survivors in clinical practice: a systematic review. Breast Cancer Res Treat.

[6] Dumas E., Jochum F., Coussy F. (2025). Explaining the relationships between age, endocrine therapy persistence, and risk of recurrence in hormone receptor-positive early breast cancer: a nationwide cohort study. J Clin Oncol.

[7] Hershman D.L., Shao T., Kushi L.H. (2011). Early discontinuation and non-adherence to adjuvant hormonal therapy are associated with increased mortality in women with breast cancer. Breast Cancer Res Treat.

[8] Ribi K., Luo W., Bernhard J. (2016). Adjuvant tamoxifen plus ovarian function suppression versus tamoxifen alone in premenopausal women with early breast cancer: patient-reported outcomes in the suppression of ovarian function trial. J Clin Oncol.

[9] Early Breast Cancer Trialists' Collaborative Group (EBCTCG) (2022). Aromatase inhibitors versus tamoxifen in premenopausal women with oestrogen receptor-positive early-stage breast cancer treated with ovarian suppression: a patient-level meta-analysis of 7030 women from four randomised trials. Lancet Oncol.

[10] Bernhard J., Luo W., Ribi K. (2015). Patient-reported outcomes with adjuvant exemestane versus tamoxifen in premenopausal women with early breast cancer undergoing ovarian suppression (TEXT and SOFT): a combined analysis of two phase 3 randomised trials. Lancet Oncol.

[11] Kennedy-Martin T., Curtis S., Faries D., Robinson S., Johnston J. (2015). A literature review on the representativeness of randomized controlled trial samples and implications for the external validity of trial results. Trials.

[12] Barlow L., Westergren K., Holmberg L., Talback M. (2009). The completeness of the Swedish Cancer Register: a sample survey for year 1998. Acta Oncol.

[13] Emilsson L., Lindahl B., Koster M., Lambe M., Ludvigsson J.F. (2015). Review of 103 Swedish healthcare quality registries. J Intern Med.

[14] Ludvigsson J.F., Andersson E., Ekbom A. (2011). External review and validation of the Swedish national inpatient register. BMC Public Health.

[15] Nystrom L., Larsson L.G., Rutqvist L.E. (1995). Determination of cause of death among breast cancer cases in the Swedish randomized mammography screening trials. A comparison between official statistics and validation by an endpoint committee. Acta Oncol.

[16] Wallerstedt S.M., Wettermark B., Hoffmann M. (2016). The first decade with the Swedish prescribed drug register - a systematic review of the output in the scientific literature. Basic Clin Pharmacol Toxicol.

[17] von Elm E., Altman D.G., Egger M. (2008). The Strengthening the Reporting of Observational Studies in Epidemiology (STROBE) statement: guidelines for reporting observational studies. J Clin Epidemiol.

[18] Reddy J., Oktay K. (2012). Ovarian stimulation and fertility preservation with the use of aromatase inhibitors in women with breast cancer. Fertil Steril.

[19] Lambertini M., Moore H.C.F., Leonard R.C.F. (2018). Gonadotropin-releasing hormone agonists during chemotherapy for preservation of ovarian function and fertility in premenopausal patients with early breast cancer: a systematic review and meta-analysis of individual patient-level data. J Clin Oncol.

[20] Cramer J.A., Roy A., Burrell A. (2008). Medication compliance and persistence: terminology and definitions. Value Health.

[21] Sweden RCCR (2025). Uppföljning av cancervården (Follow-up of cancer care)-RCC Kunskapsbanken. https://kunskapsbanken.cancercentrum.se/diagnoser/brostcancer/vardprogram/uppfoljning-av-cancervarden/.

[22] Zhang S., Liu Q., Chang M. (2023). Chemotherapy impairs ovarian function through excessive ROS-induced ferroptosis. Cell Death Dis.

[23] Ludvigsson J.F., Appelros P., Askling J. (2021). Adaptation of the Charlson comorbidity index for register-based research in Sweden. Clin Epidemiol.

[24] Tolaney S.M., Garrett-Mayer E., White J. (2021). Updated standardized definitions for efficacy end points (STEEP) in adjuvant breast cancer clinical trials: STEEP version 2.0. J Clin Oncol.

[25] Royston P., Parmar M.K. (2002). Flexible parametric proportional-hazards and proportional-odds models for censored survival data, with application to prognostic modelling and estimation of treatment effects. Stat Med.

[26] Akaike H. (1981). Likelihood of a model and information criteria. J Econom.

[27] Stensrud M.J., Hernan M.A. (2020). Why test for proportional hazards?. JAMA.

[28] Early Breast Cancer Trialists' Collaborative Group (EBCTCG) (2005). Effects of chemotherapy and hormonal therapy for early breast cancer on recurrence and 15-year survival: an overview of the randomised trials. Lancet.

[29] Bright E.E., Finkelstein L.B., Nealis M.S. (2023). A systematic review and meta-analysis of interventions to promote adjuvant endocrine therapy adherence among breast cancer survivors. J Clin Oncol.

[30] Yussof I., Mohd Tahir N.A., Hatah E., Mohamed Shah N. (2022). Factors influencing five-year adherence to adjuvant endocrine therapy in breast cancer patients: a systematic review. Breast.

[31] Reeder-Hayes K.E., Mayer S.E., Lund J.L. (2021). Adherence to endocrine therapy including ovarian suppression: a large observational cohort study of US women with early breast cancer. Cancer.

[32] Sukumar J.S., Quiroga D., Kassem M. (2021). Patient preferences and adherence to adjuvant GnRH analogs among premenopausal women with hormone receptor positive breast cancer. Breast Cancer Res Treat.

[33] Villarreal-Garza C., Bargallo-Rocha J.E., Soto-Perez-de-Celis E. (2016). Real-world outcomes in young women with breast cancer treated with neoadjuvant chemotherapy. Breast Cancer Res Treat.

[34] Sundell M., Brynhildsen J., Fredrikson M., Hoffmann M., Spetz Holm A.C. (2024). Insufficient use of menopausal hormone therapy in Swedish women with early or premature menopause caused by bilateral oophorectomy: a register-based study. BJOG.

[35] Ruddy K., Mayer E., Partridge A. (2009). Patient adherence and persistence with oral anticancer treatment. CA Cancer J Clin.

[36] Pagani O., Walley B.A., Fleming G.F. (2023). Adjuvant exemestane with ovarian suppression in premenopausal breast cancer: long-term follow-up of the combined TEXT and SOFT trials. J Clin Oncol.

[37] Pistilli B., Paci A., Ferreira A.R. (2020). Serum detection of nonadherence to adjuvant tamoxifen and breast cancer recurrence risk. J Clin Oncol.

[38] Markkula A., Hietala M., Henningson M., Ingvar C., Rose C., Jernstrom H. (2012). Clinical profiles predict early nonadherence to adjuvant endocrine treatment in a prospective breast cancer cohort. Cancer Prev Res (Phila).

[39] Schmid S.M., Eichholzer M., Bovey F., Myrick M.E., Schotzau A., Guth U. (2012). Impact of body mass index on compliance and persistence to adjuvant breast cancer therapy. Breast.

[40] Chlebowski R.T., Aiello E., McTiernan A. (2002). Weight loss in breast cancer patient management. J Clin Oncol.

[41] Todd A., Waldron C., McGeagh L. (2024). Identifying determinants of adherence to adjuvant endocrine therapy following breast cancer: a systematic review of reviews. Cancer Med.

[42] Collin L.J., Cronin-Fenton D.P., Ahern T.P. (2021). Early discontinuation of endocrine therapy and recurrence of breast cancer among premenopausal women. Clin Cancer Res.

