# Supplementary material for: Adherence to adjuvant endocrine therapy including GnRH-analogues and survival: a population-based cohort study
Source: eClinicalMedicine. 2025 Sep 12;88:103493. doi: 10.1016/j.eclinm.2025.103493 (PMC12572808; doi:10.1016/j.eclinm.2025.103493)
Supplement: Supplementary Table S1 [file mmc1.docx]

**Supplementary Table 1. Association between types of adjuvant endocrine treatment (aromatase inhibitors (AI), tamoxifen (TAM), TAM + gonadotropin-releasing hormone analogues (GnRHa), and AI + GnRHa) and non-adherence in premenopausal women stratifying on chemotherapy:**

|  | **Received chemotherapy (either neoadjuvantly or adjuvantly)** | | | **Received no chemotherapy** | | |
| --- | --- | --- | --- | --- | --- | --- |
| **Type of endocrine therapy** |  |  |  |  |  |  |
| **Crude model** | **n=2345** | | | **n=1352** | | |
|  | **OR**  **of non-adherence** | **95% CI** | **p-value** | **OR**  **of non-adherence** | **95% CI** | **p-value** |
| **TAM** | 1.00 (Ref.) |  |  | 1.00 (Ref.) |  |  |
| **TAM + GnRHa** | 1.57 | 1.24-1.98 | <0.001 | 1.53 | 0.87-2.72 | 0.142 |
| **AI + GnRHa** | 1.55 | 1.17-2.06 | 0.002 | 1.49 | 0.79-2.83 | 0.222 |
| **Adjusted model*** | **n=2332** | | | **n=1351** | | |
|  | **OR**  **of non-adherence** | **95% CI** | **p-value** | **OR**  **of non-adherence** | **95% CI** | **p-value** |
| **TAM** | 1.00 (Ref.) |  |  | 1.00 (Ref.) |  |  |
| **TAM + GnRHa** | 1.60 | 1.21-2.13 | 0.001 | 2.15 | 1.14-4.06 | 0.019 |
| **AI + GnRHa** | 1.73 | 1.25-2.42 | 0.001 | 2.04 | 1.01-4.11 | 0.046 |

*Adjusted for age, Charlson Comorbidity Index, history of urinary tract disorder, history of depression/anxiety, history of gynecological disorder, history of rheumatic disease, TNM stage, treating hospital, neoadjuvant/adjuvant chemotherapy, adjuvant radiotherapy, and year of diagnosis.
