# Supplementary material for: Adherence to adjuvant endocrine therapy including GnRH-analogues and survival: a population-based cohort study
Source: eClinicalMedicine. 2025 Sep 12;88:103493. doi: 10.1016/j.eclinm.2025.103493 (PMC12572808; doi:10.1016/j.eclinm.2025.103493)
Supplement: Supplementary Figure S1 [file mmc2.pptx]

## Slide 1
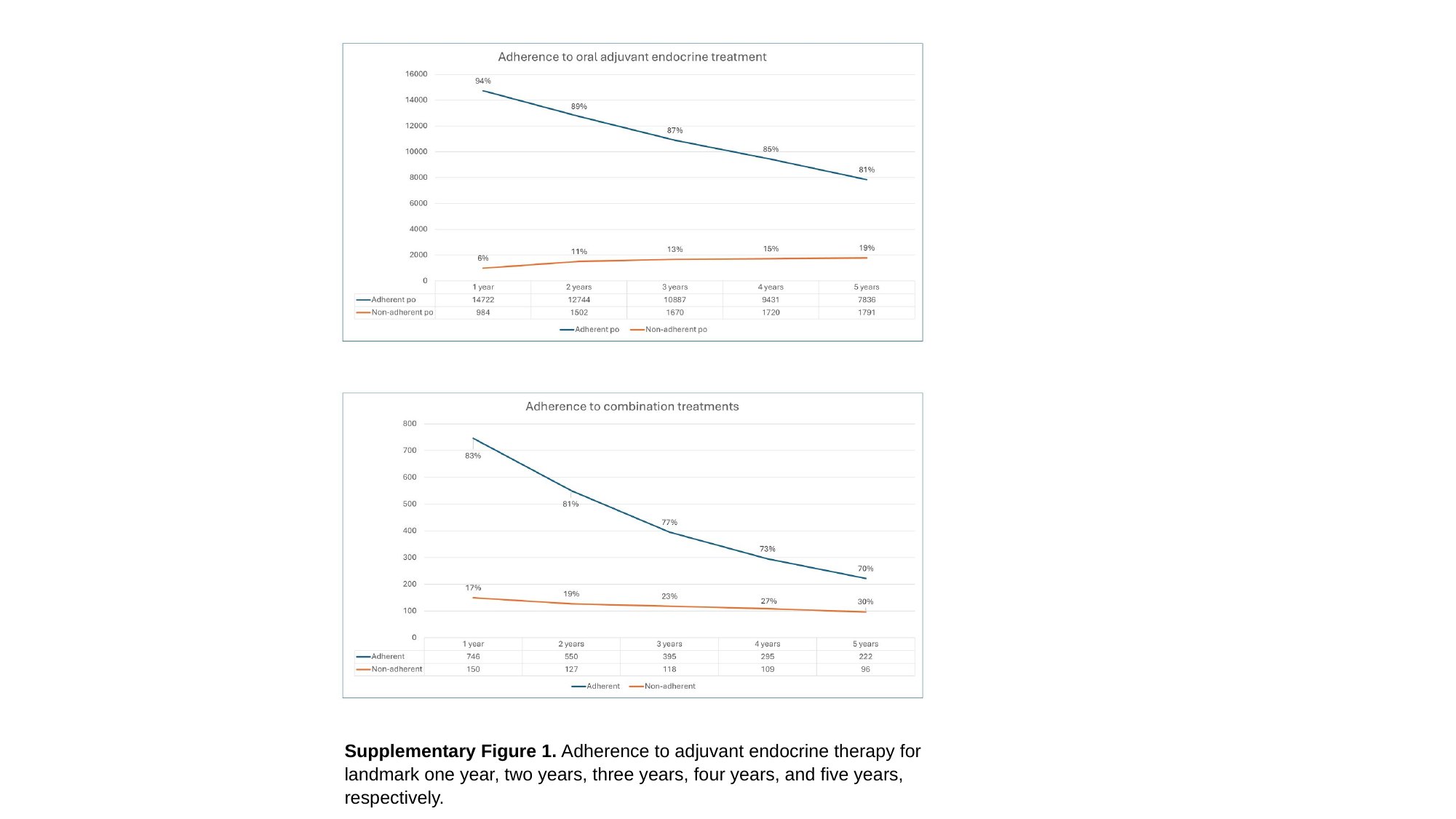

Supplementary Figure 1. Adherence to adjuvant endocrine therapy for landmark one year, two years, three years, four years, and five years, respectively.
